# *Oenococcus oeni* in Chilean Red Wines: Technological and Genomic Characterization

**DOI:** 10.3389/fmicb.2018.00090

**Published:** 2018-02-14

**Authors:** Jaime Romero, Carolina Ilabaca, Mauricio Ruiz, Carla Jara

**Affiliations:** ^1^Laboratorio de Biotecnología, Instituto de Nutrición y Tecnología de los Alimentos, Universidad de Chile, Santiago, Chile; ^2^Departamento de Agroindustria y Enología, Facultad de Ciencias Agronómicas, Universidad de Chile, Santiago, Chile; ^3^Helios Innovation, Valparaíso, Chile

**Keywords:** wine, malolactic fermentation, malolactic bacteria, *Oenococcus oeni*, terroir, genome, bacterial

## Abstract

The presence and load of species of LAB at the end of the malolactic fermentation (MLF) were investigated in 16 wineries from the different Chilean valleys (Limarí, Casablanca, Maipo, Rapel, and Maule Valleys) during 2012 and 2013, using PCR-RFLP and qPCR. *Oenococcus oeni* was observed in 80% of the samples collected. Dominance of *O. oeni* was reflected in the bacterial load (*O. oeni*/total bacteria) measured by qPCR, corresponding to >85% in most of the samples. A total of 178 LAB isolates were identified after sequencing molecular markers, 95 of them corresponded to *O. oeni*. Further genetic analyses were performed using MLST (7 genes) including 10 commercial strains; the results indicated that commercial strains were grouped together, while autochthonous strains distributed among different genetic clusters. To pre-select some autochthonous *O. oeni*, these isolates were also characterized based on technological tests such as ethanol tolerance (12 and 15%), SO_2_ resistance (0 and 80 mg l^−1^), and pH (3.1 and 3.6) and malic acid transformation (1.5 and 4 g l^−1^). For comparison purposes, commercial strain VP41 was also tested. Based on their technological performance, only 3 isolates were selected for further examination (genome analysis) and they were able to reduce malic acid concentration, to grow at low pH 3.1, 15% ethanol and 80 mg l^−1^ SO_2_. The genome analyses of three selected isolates were examined and compared to PSU-1 and VP41 strains to study their potential contribution to the organoleptic properties of the final product. The presence and homology of genes potentially related to aromatic profile were compared among those strains. The results indicated high conservation of malolactic enzyme (>99%) and the absence of some genes related to odor such as phenolic acid decarboxylase, in autochthonous strains. Genomic analysis also revealed that these strains shared 470 genes with VP41 and PSU-1 and that autochthonous strains harbor an interesting number of unique genes (>21). Altogether these results reveal the presence of local strains distinguishable from commercial strains at the genetic/genomic level and also having genomic traits that enforce their potential use as starter cultures.

## Introduction

Malolactic fermentation (MLF) is a process performed by lactic acid bacteria (LAB) that transforms malic acid into lactic acid and CO_2_, which causes a decrease in the total acidity and improvement of the taste, flavor, and microbial stability of wine (Henick-Kling, 1995; Capozzi et al., 2010). Those bacteria are naturally present in grapes, musts and wines. The predominant genera are *Leuconostoc, Pediococcus, Lactobacillus*, and *Oenococcus* (Lonvaud-Funel, 1995).

Previous studies investigating autochthonous LAB in winemaking have reported the presence of *Oenococcus oeni* strains in spontaneous MLF (Marques et al., 2011; Nisiotou et al., 2015; Cafaro et al., 2016). The genetic diversity of *O. oeni* has been shown in studies from different winemaking regions worldwide. Bartowsky et al. (2003) determined that *O. oeni* strains that originated from the same winery in Australia were either indistinguishable or closely related to each other. In Castilla-La Mancha, Spain, Cañas et al. (2009) showed that MLF was dominated by a variable number of *O. oeni* genotypes. The same observation of the genetic diversity in these bacteria was reported in La Rioja, Spain, and Apulia, Italy (González-Arenzana et al., 2012; Garofalo et al., 2015).

Bacterial dynamics during MLF have been studied using culture-dependent techniques. The major drawback of this strategy is the impossibility of correctly obtaining the diversity and dynamics of LAB during MLF (Spano et al., 2007). However, culture-independent analysis methods have been developed and are commonly used to detect and identify microorganisms directly from wine by analyzing their DNA. Ilabaca et al. (2014) designed a 16S rRNA Polymerase Chain Reaction-Restriction Fragment Length Polymorphism (16S rRNA PCR-RFLP) culture-independent strategy that was a reliable tool for the identification and differentiation of winemaking LAB strains isolated during the MLF process. González-Arenzana et al. (2012) studied LAB populations in red wine (La Rioja, Spain) and compared two strategies (culture-dependent and culture-independent methods). Both methods were complementary during MLF. However, the culture-independent methods allowed the detection of a vaster number of species than the culture-dependent methods. Therefore, *O. oeni* was the most frequently detected bacterium during MLF. The conclusions drawn from these molecular studies indicate that LAB populations are diverse during the early stages of MLF; however, *O. oeni* subsequently becomes the most dominant bacterial population during the MLF process. This result is consistent with the previous observations derived using culture-dependent approaches (Rodas et al., 2003; López et al., 2007).

The genetic characterization of *O. oeni* has permitted the evaluation of differences between *O. oeni* isolates from diverse winemaking locations using several molecular strategies, including multilocus sequence typing (MLST) (de las Rivas et al., 2004; Bilhère et al., 2009; Bridier et al., 2010; Bordas et al., 2013; Wang et al., 2015). Several studies reported a high level of allelic diversity and a combination of alleles among *O. oeni* isolates (Bilhère et al., 2009; Bon et al., 2009). However, genetic differences between *O. oeni* strains must be studied and understood because they might affect the quality of the wine, especially in terms of organoleptic properties. Recently, the genome sequences of *O. oeni* strains have been made available from different enological locations (Mills et al., 2005; Borneman et al., 2010; Capozzi et al., 2014; Lamontanara et al., 2014; Jara and Romero, 2015). Jara and Romero (2015) suggested that genomic analyses might provide insights into the adaptation of strains to wine-hostile conditions and their contributions to the organoleptic properties of the final product. Cappello et al. (2017) proposed an association between inter/intra-species diversity and bacterial metabolic traits that impacted the wine's organoleptic characteristics. Additionally, these authors showed evidence of the importance of the enzymatic potential of LAB to enrich the wine aroma.

In Chile, most MLF processes are conducted spontaneously by the resident LAB microbiota in the cellars. However, spontaneous MLF has drawbacks, including stuck fermentation and contamination by microorganisms, which risk altering the wine quality. To solve this problem, the use of microbial starters has been introduced with commercial strains isolated from other wine-producing countries. However, in Chile some of these starters have produced poor results due to their insufficient imposition during MLF (Ilabaca et al., 2014). Among the many factors that impede the development of *O. oeni*, the most important is the presence of inhibiting factors, such as a low pH, high ethanol content, and low malic acid content.

This study reports the first genetic and technological characterization of *O. oeni* strains retrieved from spontaneous MLF in different Chilean valleys. Additionally, the genomes of selected isolates were examined and compared them to PSU-1 and VP41 strains to study their potential contributions to the organoleptic properties of the final product. These characteristics could be the basis for obtaining autochthonous isolates to serve as starters capable of improving the typicity of Chilean wines.

## Materials and methods

### Samples

Spontaneous MLF samples (58) were collected in 2012 and 2013 from 16 wineries, including cultivars (cvs.) Cabernet Sauvignon and Carménère, located in four Chilean valleys: Limarí (30°38′S–71°24′W), Maipo (33°45′S–70°46′W), Rapel (34°15′S–72°00′W), and Maule (35°58′S–72°19′W), sampling four wineries per valley. All the tested wineries carried out spontaneous MFL without commercial starter; sampling was performed at the end of the MLF. The winemaking process was initiated with healthy grapes harvested from March to May, followed by the traditional vinification practices of each winery. As a general rule of each winery alcoholic fermentation (AF) using commercial freeze-dried yeast was performed stainless steel tanks at 22–25°C. Spontaneous MLF was carried out immediately after AF in stainless steel tanks at 18–22°C for 30–40 days. Samples were aseptically collected at the end of MLF, where the wines showed average chemical parameters: ethanol (14.1%v/v) and pH (3.7). The criterion for defining the end of MLF in each winery is the reduction in the content of the L-malic acid (<0.3 g/L) in the wines determined using an enzymatic test (Boehringer Manheim; Mannheim, Germany). Samples were stored at 4°C until being processed.

### Bacterial isolation

The bacterial isolation was carried out using medium for *Leuconosctoc oenos* (MLO), following indications by Blasco et al. (2003). This medium was supplemented with 2 ml L^−1^ sodium azida (Winkler, Chile) and 3 ml L^−1^ cyclohexamide (Sigma-Aldrich) to eliminate yeasts and acid acetic bacteria, respectively (Ruiz et al., 2008). Serial dilutions were plated onto the MLO media and incubated for 5–7 days at 28°C, under anaerobic conditions. After count colonies (CFU mL^1^) 10 colonies per sample were randomly chosen. This selection was realized according to the phenotypic characterization (Mesas et al., 2011). Each selected colony was transferred and purified through two rounds of streak plating onto fresh agar plates. The isolates were maintained in a cryobank at −80°C.

### Reference and commercial strains

Lactic acid bacteria commercial strains for comparison in genetic diversity study were used. This included Lallemand (Lalvin VP41®, Lalvin 31®, uvaferm Alpha®, uvaferm Beta®, Lalvin Elios®, PN4®, Lalvin MTO®); Lamothe Abiet (Oeno 1, Oeno 2); Laffort (Lactoenos B28 PreAc®). Lalvin VP41® was included in the technological evaluation.

### DNA extraction from wine

The initial step for our culture independent approach was the extraction of DNA directly from wine with MLF, according to Jara et al. (2008) and Ilabaca et al. (2014). This DNA was used to quantify bacterial load (see below).

### DNA extraction from bacterial isolates

In the case of LAB isolates, each of the colonies selected was suspended in 200 mL PBS, with vigorous agitation, followed by centrifugation at 5,000 × g for 5 min. Subsequently, 20 μL aliquot of 20 mg mL^−1^ Lysozyme (Sigma) was added to the pellets, which were subsequently incubated at 37°C for 30 min. The Wizard Genomic DNA Purification Kit (Promega) was used for DNA extraction according to the protocol for isolating genomic DNA from Gram-positive bacteria the manufacturer's instructions. DNA obtained was frozen at −20°C until processed. The identification de LAB from samples MLF wine was done by 16S rRNA PCR-RFLP, according to Ilabaca et al. (2014).

### Amplification *rpo*B gen

To distinguish *O. oeni* isolates, RNA polymerase ß subunit (*rpo*B) were PCR amplified using methodology described by Bridier et al. (2010). DNA sequencing was performed by Macrogen (USA) and the analyses were done by BLAST (basic Alignment Search Tool). From the *rpo*B sequence results of autochthonous *O. oeni* isolates and commercial strain (VP41), were analyzed with its differences about nucleotides among them by used BioEdit version 7.1.9, generating signature groups (Drancourt and Raoult, 2002). The signature sequences corresponded to part of a coding sequence of a gene which, because it is shared by different isolates, is thought to be evolutionarily conserved and therefore can serve to trace taxonomic relationships among these isolates (Gupta, 1998).

### Quantitative PCR (qPCR) and total bacteria and *O. oeni*

Both total bacteria and load of *O. oeni* were quantified by qPCR reactions based on detection of SYBR fluorescence. The qPCR reactions were performed using an AriaMx real-time PCR System (Agilent Technologies), using primers and programs described in Table 1. PCR amplification was performed in 10 μL of mix containing 1 μL of DNA 0.5 *p*mol/mL of each respective primer 5 μL of LightCycler 480 SYBR Green I Master (Roche) and 3.5 μL of Milli-Q sterile H_2_O. All samples were analyzed in triplicate. The statistical analyses among bacterial loads valleys were determined by ANOVA using R Development Core Team (2008), and the *post-hoc* test was performed by pairwise.t.test.

**Table 1:** Primers and programs for quantitative PCR

	**Programs**	**Primer**	**Sequences 5′-3′**	**References**
Total bacteria	95°C, 5 m; 95°C, 5 s; 55°C, 10 s; 72°C, 10 s	341	CCTACGGGAGGCAGCAG	Opazo et al., 2012
		788	GGACTACCAGGGTATCTAA	
*Oenococcus oeni*	95°C, 5 m; 95°C, 10 s; 55°C, 10 s; 72°C, 10 s	RpoB F	CGATATTCTCCTTTCTCCAATG	Bridier et al., 2010
		RpoB R	CTTTAGCGATCTGTTCCAATG	

### Multilocus sequence typing (MLST) analysis

Based on *rpo*B gen analysis (signature groups), the autochthonous *O. oeni* isolates were selected about two criteria. First, the different *rpo*B sequences to obtain maximal diversity. Second, *rpo*B sequences isolates from different Chilean Valleys.

To analysis of MLST seven housekeeping genes for this study were selected. These genes were *gyrB* (Gyrase Beta subunit), *g6pd* (Glucose-6-phosphate dehydrogenase), *mleA* (Malolactic enzyme), *pgm* (Phosphoglucomutase), *dnaE* (DNA polymerase III, alfa subunit), *pgm* (Phosphorybosylaminoimidazole), *purK* (Phosphoribosylamino-imidazole carboxylase), *rpoB* (RNA polymerase, Beta subunit). The *recP* gene was not included in our analysis because it was not present in our isolates. After the examination of a subset of 114 *O. oeni* genomes originated from diverse geographic locations, only 40 strains harbored this gene (Supplementary Table S1). The PCR mixes were performed according to de las Rivas et al. (2004) and Bridier et al. (2010).

The PCR products were sequenced by Macrogen (USA). The analysis of sequences obtained by MLST was realized using BioEdit version 7.1.9 software and a dendrogram was constructed by UPGMA (unweighted pair-group method with arithmetic mean) method. The evolutionary distances were computed using the Maximum Composite Likelihood method, using the Mega 6 (version 6.0) software from the website (Tamura et al., 2013).

The sequences of each gen of autochthonous *O. oeni* isolates selected and commercial strain (VP41), were analyzed with its differences about nucleotides among them by used BioEdit version 7.1.9. The base pair of each gen analyzed were *rpoB gen* 579 bp; *dnaE* gen 641 bp; *g6pd* gen 591 bp; *gyrB* gen 544 bp; *mleA* gen 355 bp; *pmg* gen 580 bp; *purK* gen 493 bp.

### Evaluation of technological properties of *O. oeni* isolates

Based on the MLST analysis, some strains were included in the technological tests. For the different technological tests, autochthonous *O. oeni* isolates were grown in MLO broth to early stationary phase. An inoculum of 1^*^10^8^ cells mL^−1^ of each *O. oeni* isolates was used to inoculate wine-like medium (Bordas et al., 2015). Our criterion to select the isolates was a first test; wine-like medium was supplemented with two ethanol concentrations (12% v/v and 15% v/v), the isolates natives were incubated at 25°C for 10 days. Then, the best isolates were submitted to wine-like medium supplemented with malic acid 1.5 and 4g L^−1^ at 25°C for 10 days. Wine-like medium either at pH 3.1 and 3.6 and were incubated at 25°C for 24 h and 5 days. Finally, the isolates were incubated into this medium, utilizing potassium metabisulfite concentrations (0 and 80 ppm free SO_2_) at 25°C for 7 days. All bacterial growth was per triplicate and monitored by culture dependent analyses. The significant differences among isolates of each tests were determined by Kruskal–Wallis test using R Development Core Team (2008), and the *post-hoc* test was performed by posthoc.kruskal.dunn.test belonging to the PMCMR package.

### Genomic analyses

Based on technological characteristics, the draft genome of three autochthonous *O. oeni* isolates were obtained and analyzed. Genome characteristics and the accession numbers about those isolates have been previously reported (Jara and Romero, 2015). Using the online tool at bioinformatics.psb.ugent.be, a Venn diagram was generated to compared genome composition of *O. oeni* autochthonous isolates with 2 reference strains: VP41 (ACSE00000000) and PSU-1 (NC_008528).

Three approaches to calculate average nucleotide identity from genome sequences of O. *oeni* autochthonous isolates and 2 reference strains: VP41 (ACSE00000000) and PSU-1 (NC_008528) were used. Those were: DNA–DNA hybridization (DDH), Average Nucleotide Identity (ANI) and Orthology (OrthoANI). The similarity obtained by DNA–DNA hybridization (DDH) to genome-sequence-based similarity according to Meier-Kolthoff et al. (2013) was determined. Second approach utilized was average nucleotide identity (ANI) according to Rosselló-Mora (2006). Finally, third approach was average nucleotide identity by orthology (OrthoANI) according to Lee et al. (2016).

The presence and homology of 21 (*abf*, *arcA, alsS, alsD, arcB, arcC, bgl, citD, citE, citF, estA, estB, estC, gshR, maeP, metB, metC, metK, mleA, pad, prtP)* genes potentially related to aromatic profile were analyzed from *O. oeni*, autochthonous isolates and reference strains VP41 and PSU-1 genome sequences. These genes were taken from literature (Sumby et al., 2009, 2013; Mtshali et al., 2012; Cappello et al., 2014). The orthologous clustering analysis were performed using Inparanoid (Fouts et al., 2012; Sonnhammer and Östlund, 2015). To analyze enzymes groups (glycosidases, esterases, proteases, citrate metabolism, and peptidases), that may play a role in the wine organoleptic properties into proteomes of *O. oeni*, autochthonous isolates and reference strains VP41 and PSU-1 genome sequences were realized using coding sequence for protein (CDS) by PfamScan (Finn et al., 2016).

## Results

### Lactic acid bacteria in different chilean valleys

A total of 60 wine samples from four Chilean valleys were used to characterize the bacterial population dynamics at the end of spontaneous MLF with 16S rRNA PCR-RFLP. *O. oeni* was observed in 80% of the wine samples. *Lactobacillus* and *Leuconostoc* were detected in 4.5% and 2.3% of the samples, respectively. Furthermore, *Oenococcus*/*Pediococcus* and *Oenococcus/Leuconoctoc* were found together at frequencies of 4.5 and 2.3%, respectively.

**Figure 1:**
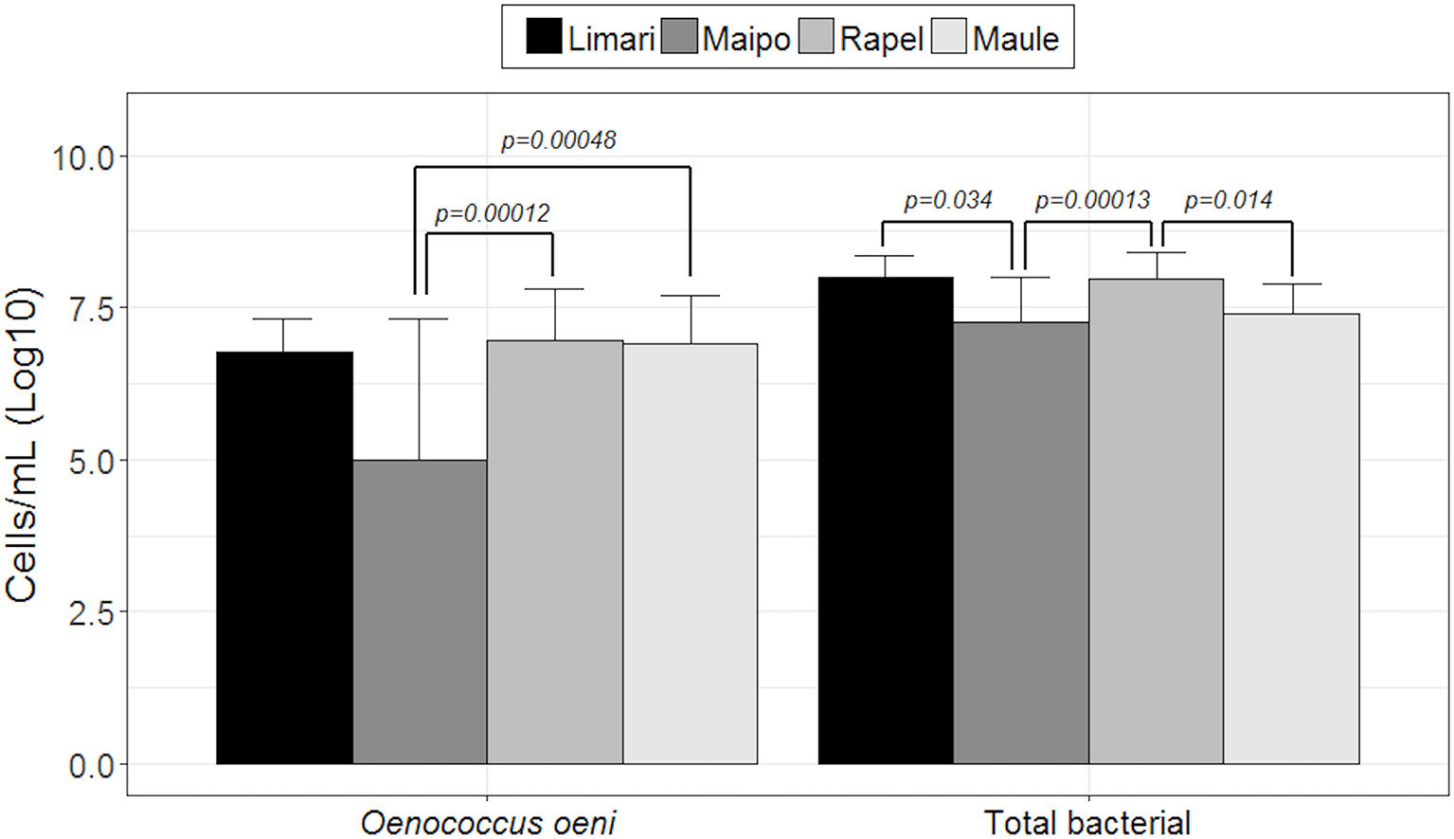
Total bacterial and *O. oeni* load quantified by qPCR in wine from spontaneous MLF different valleys examined (log_10_ scale).

Both the total bacterial and *O. oeni* loads were explored by qPCR. Figure 1 shows the number of microorganisms (log_10_ scale) represented by each valley ordered from north to south (Limarí, Maipo, Rapel, and Maule). Limarí and Rapel valleys showed the highest total bacterial load with 10^7^ total bacteria per mL of wine. Significant differences were found in total bacterial loads among valleys; Limarí and Maipo valleys (*p*-value: 0.034); Maipo and Rapel valleys (*p*-value: 0.000013); Rapel and Maule valleys (*p*-value: 0.0014). On the other hand, Rapel and Maule valleys, showed the highest *O. oeni* loads, with 10^6^ *O. oeni* per mL of wine. Significant differences were found in *O. oeni* load among valleys, Maipo and Maule valleys (*p*-value: 0.00048) and Maipo and Rapel valleys (*p*-value: 0.00012). In summary, the *O. oeni* loads and the total bacterial loads indicated a dominance of *O. oeni* at the end of MLF.

### Identification and characterization of LAB isolates

**Figure 2:**
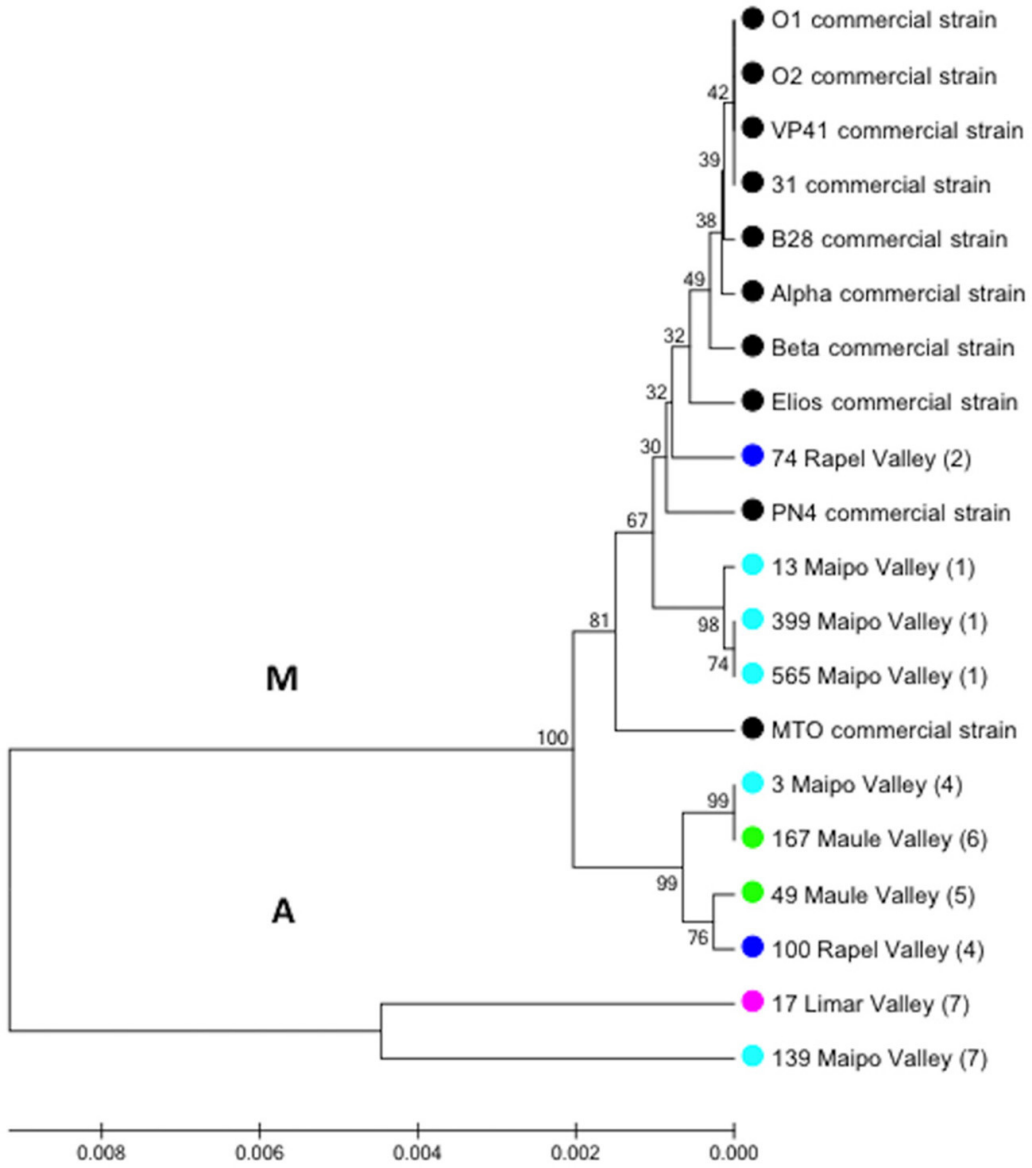
Phylogenetic reconstruction based on the seven gene markers MLST scheme. Analysis included *O. oeni* commercial strains and Chilean autochthonous *O. oeni* isolates, which were distributed in two genetic groups indicated as M and A. Colored circles indicate the origin of each isolate (Chilean valley or commercial). Numbers correspond to signature group described in Table 2.

A total of 158 autochthonous LAB isolates were retrieved from red wine collected at the end of spontaneous MLF in wineries located in the Limarí, Maipo, Rapel, and Maule Valleys and characterized using phenotypic tests (catalase and Gram staining). Among them, 75 strains were identified as *O. oeni* by PCR-*rpo*B sequencing. The analysis of a 579-bp *rpo*B sequence generated signature groups according to the different nucleotides and positions using the *rpo*B sequence from the VP41 reference strain. Based on this analysis, 46 autochthonous *O. oeni* isolates were found to differ from VP41, and 7 signature groups were observed. The signature groups are described in detail in Table 2, including the signature, number of isolates and origin. Based on these signature groups, 10 autochthonous *O. oeni* isolates for analysis using MLST typing were selected. The selection was based on two inclusion criteria of at least 1 isolate from each signature group and two strains per valley. Additionally, 10 *O. oeni* commercial strains were included in the MLST genetic analysis for comparison. Concatenation of the sequences for each of the seven genes formed a 3,783-bp sequence, which was examined using the MEGA software. The resulting dendrogram (Figure 2) showed two major genetic groups of *O. oeni* (M and A). Group M included all of the commercial strains and some autochthonous *O. oeni* isolates (13, 399, 565, and 74). Furthermore, the commercial strain MTO presented a transposon in *purK* from 423 to 1,282 bp. In contrast, group A was only composed of autochthonous *O. oeni*. Group M was composed of two subgroups that showed different distributions of autochthonous isolates based on the signature groups. Interestingly, two isolates obtained from geographically separated valleys (500 kilometers) grouped together in A.

**Table 2:** Signature groups of autochthonous *O. oeni* isolates from different valleys.

**Valleys**	**Different nucleotides in *rpo*B gen**	**Different position in *rpo*B gen**	**Number of signature groups**
Maipo	G	23	1
Maipo	G	23	1
Maipo	G	23	1
Rapel	G	23	1
Rapel	G	23	1
Rapel	G	23	1
Rapel	G	23	1
Rapel	G	23	1
Maipo	G	23	1
Maipo	G	23	1
Maipo	G	23	1
Maule	G	23	1
Maule	G	23	1
Maipo	G	23	1
Rapel	G	536	2
Maipo	GT	23/43	3
Maipo	CTGT	38/116/234/347	4
Rapel	CTGT	38/116/234/347	4
Rapel	CTGT	38/116/234/347	4
Maule	CTGT	38/116/234/347	4
Maipo	CTGT	38/116/234/347	4
Maule	CTGT	38/116/234/347	4
Maipo	CTGT	38/116/234/347	4
Maipo	CTGT	38/116/234/347	4
Rapel	CTGT	38/116/234/347	4
Rapel	CTGT	38/116/234/347	4
Maule	CTGT	38/116/234/347	4
Maule	CTGT	38/116/234/347	4
Maule	CTGT	38/116/234/347	4
Maipo	CTGT	38/116/234/347	4
Maipo	CTGT	38/116/234/347	4
Maipo	CTGT	38/116/234/347	4
Rapel	CTGT	38/116/234/347	4
Rapel	CTGT	38/116/234/347	4
Maipo	CTGT	38/116/234/347	4
Maipo	CTGT	38/116/234/347	4
Maule	CTGT	38/116/234/347	4
Maule	CTGT	38/116/234/347	4
Maipo	CTGT	38/116/234/347	4
Rapel	CTGT	38/116/234/347	4
Rapel	CTGT	38/116/234/347	4
Maule	GTTTGT	38/42/43/116/234/347	5
Maipo	CGTGCCTGATTTTGCCAGTACCAGT CCAGTAAATATCCGCTGATCGTG	26/36/53/59/85/89/116/137/140/154/167/215/224/260/266/267/281/299/ 247/350/351/362/363/365/368/377/392/425/443/480/482/485/ 488/489/491/594/502/503/509/515/518/521/524/533/534/535/ 536/ 557	6
Limarí	CGTGCCTGATTTTGCCAGTACCA GTCCAGTAAATATCCGCTGATCGTG	26/36/53/59/85/89/116/137/140/154/167/215/224/260/266/267/281/299/ 247/350/351/362/363/365/368/377/392/425/443/480/482/485/ 488/489/491/594/502/503/509/515/518/521/524/533/534/535/ 536/ 557	6
Maipo	CGTGCCTGATTTTGCCAGTACCAGT CCAGTAAATATCCGCTGATCGT	26/36/53/59/85/89/116/137/140/154/167/215/224/260/266/267/281/299/ 247/350/351/362/363/365/368/377/392/425/443/480/482/485/ 488/489/491/594/502/503/509/515/518/521/524/533/534/535/ 536	7
Maipo	CGTGCCTGATTTTGCCAGTACCAG TCCAGTAAATATCCGCTGATCGT	26/36/53/59/85/89/116/137/140/154/167/215/224/260/266/267/281/299/ 247/350/351/362/363/365/368/377/392/425/443/480/482/485/ 488/489/491/594/502/503/509/515/518/521/524/533/534/535/ 536	7

### Technological properties of autochthonous *O. oeni* isolates

The ability of autochthonous strains to resist wine-like medium supplement with ethanol (12% v/v) was compared with strain VP41 (LABc). Three isolates were discarded because they were unable to survive in this medium. Therefore, only seven isolates were examined to assess their technological properties, including the kinetics of the transformation of malic acid, ethanol resistance, pH resistance, and SO_2_ resistance. Supplementary Figures S1A,B shows the ability of the isolates to degrade malic acid at two initial concentrations. The strains 139 and 565 showed the best reduction of this acid. Similarly, all strains grew in the presence of 12% ethanol, but 139 showed the best survival in 12 and 15% ethanol (Supplementary Figures S1C,D). To study the association between bacterial growth and pH tolerance, autochthonous strains were examined in media with different pH values (3.1 and 3.6). Supplementary Figures S1E,F, showed that strain 17 and 74 did not survive these conditions; in contrast, strain 139 presented the highest potential for growth in restrictive pH media. Similarly, the effect of sulfur oxide (0 and 80 ppm) was examined (Supplementary Figures S1G,H). Sulfur oxide was deleterious for strains 17, 74, and 167 whereas isolate 139 presented a high viable count over the 10-day period. In summary, the best strains according to their technological properties were 139 and 565.

### Genome-based phylogeny and genome comparison

**Figure 3:**
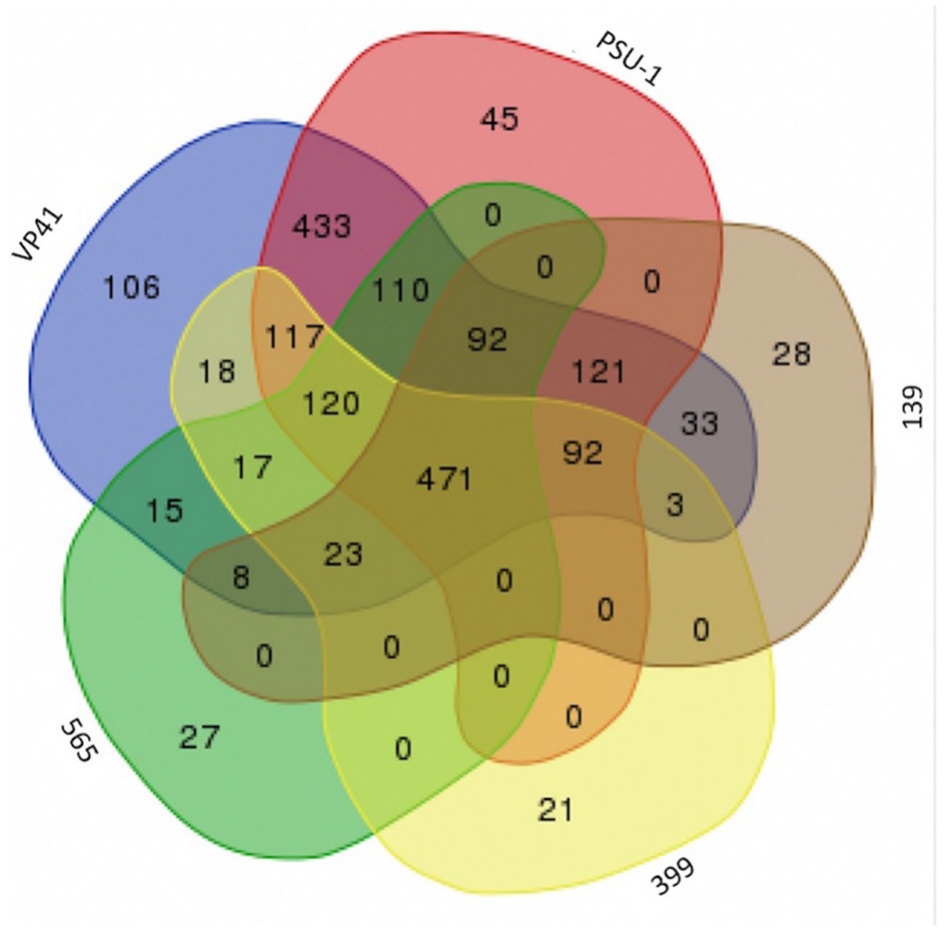
Venn diagram between genome of Chilean autochthonous *O. oeni* isolates compared with two reference strains VP41 and PSU-1.

To examine the relationships among the autochthonous *O. oeni* strains (565, 399, and 139) and the reference strains (VP41 and PSU-1), genome analyses were performed. First, the number of common genes shared by these *O. oeni* strains was evaluated; the results are shown in a Venn diagram in Figure 3. This figure revealed 470 common genes, of which 63% were uncharacterized proteins and 9% were ribosomal genes. Each strain presented unique genes, indicating that the autochthonous strains harbored an interesting number of unique genes. Strain 139 presented 28 unique genes, strain 565 presented 27 unique genes and strain 399 presented 21 unique genes. These genes encoded ABC transporters; galactose metabolism; phosphostransferase system (PTS); pentose phosphatase pathway; starch and sucrose metabolism; two component system; nicotinate and nicotinamide metabolism that might improve bacterial performance in the wine environment. Then, the autochthonous *O. oeni* genomes were compared to the reference strains using 3 methodologies (ANIb, DDH, and OrthoANI) (Table 3). The three methods showed that strain 139 had the lowest similarity to VP41 and PSU-1.

**Table 3:** Results of DDH, ANIb, and OrthoANI algorithms of autochthonous *O. oeni* genomes compared to reference strain VP41.

	**DDH**	**ANIb**	**OrthoANI**
***O. oeni***	**AWRI429 or VP41 (reference)**	**AWRI429 or VP41 (reference)**	**AWRI429 or VP41 (reference)**
139	88,5	98,29	98,5702
399	96,7	99,38	99,5249
PSU-1	96,9	99,41	99,5289
565	96,5	99,3	99,5152

### Genes that contribute to organoleptic properties

To study the potential contributions to the organoleptic properties of the final product, genes related to the improvement of the wine's aromatic profile were analyzed. The autochthonous *O. oeni* genomes were compared to reference strains in terms of families and domains of interest for the enzymes that contributed to the organoleptic properties, such as esterases, glycosidases, enzymes involved in citrate metabolism, peptidases and proteases (Table 4). Our results showed that strain 139 presented a higher number of enzymes than strains 399 and 565.

**Table 4:** Occurrence of esterases, glycosidases, citrate metabolism, peptidases, and proteases enzymes of autochthonous *O. oeni* genomes compared with reference strains (VP41 and PSU-1).

	**Reference strains**		**Autochthonous** ***O. oeni*** **isolates**		
**Enzymes**	**VP41**	**PSU-1**	**139**	**399**	**565**
Glycosidases	28	18	10	6	10
Esterases	2	2	1	1	1
Citrate met	4	4	4	4	4
Peptidases	47	44	22	17	21
Proteases	3	1	1	1	1

The genome analysis screened for the presence and identity of 21 genes encoding enzymes potentially related to the aromatic profile of the wine as previously described (Mtshali et al., 2010; Cappello et al., 2014). Table 5 showed that strain 139 harbored 14 of the 21 screened genes, which was higher than the numbers found for strains 565 and 399. Furthermore, the phenolic acid carboxylase gene (*pad*), carbamate kinase gene (*arcC*) and protease *ptrP* gene were absent in the autochthonous *O. oeni* genomes.

**Table 5:** Identities of aromatic genes found between autochthonous *O. oeni* genomes compared with reference strains (VP41 and PSU-1).

**Aromatic Genes**	**Specie**	**NCBI/UNIPROT ACC. NUM**.	**Reference strain**		***O. oeni*** **a isolates**			**Coded protein**
			**PSU-1**	**VP41**	**139**	**399**	**565**	
*mleA*	*Oenococcus oeni*	AAV65766.1	99,82	99,82	99,82	99,82	99,82	*mleA* Malolactic enzyme
*alsS*	*Oenococcus oeni*	AEW09411.1	99,64	99,64	99,64	99,82	99,82	*alsS* alfa-Acetolactate synthase
*alsD*	*Oenococcus oeni*	AEW09410.1	100	100	0	99,58	0	*alsD* alfa-Acetolactate descarboxylase
*citD*	*Oenococcus oeni*	CITD_OENOB	100	100	100	0	100	*citD* Citrate lyase g-subunit
*citE*	*Oenococcus oeni*	W5XLJ3_OENOE	99,01	99,01	100	0	0	*citE* Citrate lyase β-subunit
*citF*	*Oenococcus oeni*	A0NL52_OENOE	99,61	99,61	99,61	99,61	99,61	*citF* Citrate lyase a-subunit
*maeP*	*Oenococcus oeni*	AEW09418.1	100	100	98,78	99,69	99,69	*maeP* Putative citrate transporter
*bgl*	*Oenococcus oeni*	AIZ50378.1	99,32	99,46	99,05	0	0	*bgl* ß Glucosidase - related glycosidase
*abf*	*Oenococcus oeni*	ADJ95768.1	0	99,67	100	0	0	*abf*, a-L-arabinofuranosidase
*estA*	*Oenococcus oeni*	AFV75079.1	100	100	99,24	99,24	99,24	*estA* Predicted esterase
*estB*	*Oenococcus oeni*	AFV75078.1	99,01	99,01	99,67	98,64	98,64	*estB* Predicted esterase
*estC*	*Oenococcus oeni*	AFV75077.1	26,79	95,91	23,74	95,7	95,7	*estC* Predicted esterase
*metB*	*Oenococcus oeni*	R4HZQ9_OENOE	99,21	99,21	99,21	0	41,48	*metB* Cystathionine g-lyase
*metC*	*Oenococcus oeni*	AEW09413.1	100	100	47,31	0	39,89	*metC* Cystathionine β-lyase
*metK*	*Oenococcus oeni*	AEW09412.1	100	100	100	0	0	*metK* S-adenosylmethionine synthase
*gshR*	*Oenococcus oeni*	AEW09415.1	100	100	99,78	99,78	99,78	*gshR* Glutathione reductase
*arcA*	*Oenococcus oeni*	ARCA_OENOB	100	99,38	98,55	0	0	*arcA* Arginine deiminase
*arcB*	*Oenococcus oeni*	OTCC_OENOE	29,57	100	29,79	29,03	29,03	*arcB* Ornithine Transcarbamylase
*arcC*	*Oenococcus oeni*	ARCC_OENOE	0	100	0	0	0	*arcC* Carbamate kinase
*pad*	*Lactobacillus plantarum*	AAC45282.1	0	0	0	0	0	*pad p*henolic acid decarboxylases
*prtP*	*Lactobacillus plantarum*	CAT14096.1	0	0	0	0	0	p*rtP* Proteinase

Genes linked to increased esters and ethyl esters that contributed to the wine's fruity aromas (*estA, estB, estC*, and *metB*) were present in the autochthonous *O. oeni* genomes. Genes linked to dyacetil, acetoin, butanediol, and acetate via citrate metabolism *(citE, citF, citD maeP*, and *alsA*) were present in isolate 139, but some of these genes were absent in the other autochthonous *O. oeni* genomes (565 and 399). Genes related to odorless non-volatile glycosides and glycosidase activities that contributed to the wine aroma (*bgl* and *abf*) were present in strain 139.

## Discussion

One of the main objectives of this study was to analyze the load and diversity of LAB in wine produced with spontaneous MLF in Chilean valleys and to pre-select future starter cultures. This study covered an extensive Chilean enological region from 30°39′S to 35°50′S latitude and analyzed the total and lactic acid bacterial loads using culture-independent methodology. Our LAB screening results showed the highest prevalence *O. oeni*, which was coincident with reports from other countries (González-Arenzana et al., 2012; Pramateftaki et al., 2012; Cappello et al., 2017). Additionally, combinations of LAB, such as *O. oeni/Leuconostoc* and *O. oeni/Pediococcus* were found, which were coincident with the findings of Renouf et al. (2007), Pérez-Martín et al. (2015) and Miranda-Castilleja et al. (2016). However, the existence of *Pediococcus* in wine samples needs consideration, because these bacteria have been associated with ropiness of wine (Dols-Lafargue et al., 2008).

Furthermore, we found a predominance of the *O. oeni* load compared to the total bacterial load in all samples by qPCR analysis of the *rpo*B gen. This gene has been used for the description of LAB in fermentative environments (i.e., Renouf et al., 2006; Miranda-Castilleja et al., 2016 used the *rpo*B gene to study the dynamics and diversity of LAB in different cellars). Therefore, quantification of the bacterial DNA using the *rpo*B gen showed that this gene could be used as a marker of the *O. oeni* load and thus might be useful for monitoring MLF. However, the total bacterial and *O. oeni* loads were similar among the Limarí and Maule Valleys, which were located ~760 km apart. The influence of the local bacterial diversity on wine elaboration and the peculiar characteristics provide a local product fingerprint, as reported by Bokulich et al. (2014). Furthermore, rpoB was useful to obtain a prior genetic screening of the strains, since the sequencing of this gene allow us to distinguish between autochthonous and commercial *O. oeni* strains. This approach has been reported previously in other Gram positive bacteria such as *Staphylococcus* (Drancourt and Raoult, 2002).

Genetic typing examined using MLST evidenced the existence of two major phylogenetic clusters. Interestingly, half of the autochthonous isolates could be distinguishable from the commercial isolates, whereas the other half grouped together with the commercial strains. Within group M, the commercial strain MTO presented insertion of a transposon element in *purK*, which is one of the most interesting loci to analyze the genetic variability of the O. oeni strains (González-Arenzana et al., 2013). This insertion was reported in 7 *O. oeni* strains from Champagne, Burgundy, and Jura, France, 1 strain from Italy (Bridier et al., 2010) and 2 strains from Pineau and Jura, France (Bilhère et al., 2009). According to the MLST results, low genetic diversity among the autochthonous *O. oeni* isolates were found, which might be related to the use of housekeeping genes that could be under restricted variation. Other explanations are the exchange of DNA among *O. oeni* strains, as proposed by de las Rivas et al. (2004), where a favorable environment for horizontal gene transfer could be created in the fermentation tank/barrel. Dicks (1994) and Zúñiga et al. (1996) showed that *O. oeni* was able to receive foreign DNA by transformation *in vitro* and by conjugation. Interestingly, Campbell-Sills et al. (2015) reported that *O. oeni* isolated from MLF grouped together in a phylogenomic analysis and that strains outside this genetic group were absent during MLF.

Spontaneous MLF has drawbacks, including stuck fermentation and contamination by microorganisms, which risk altering the wine quality. To solve this problem, the use of microbial starters has been introduced with commercial strains isolated from other wine-producing countries. However, starter strains selected from the wine native microbiota of each region have better natural adaptation to the wine and maintain regional typicity (Zapparoli et al., 2003; Izquierdo et al., 2004). Ethanol and acidic environments are determinant factors for the growth of *O. oeni* in wine (Liu et al., 2014). In this context, strains able to tolerate 15% v/v ethanol and a pH of 3.1 were obtained. These results were different from the reports of Capozzi et al. (2010); Solieri et al. (2010) and Lerm et al. (2011), which showed that *O. oeni* strains were unable to survive in high ethanol concentrations (13% v/v). Strain 139 had high growth in 15% v/v ethanol and at a pH of 3.1 and exhibited high malolactic activity; these results suggested that this strain (139) adapted better to the wine environment than the other two strains (565 and 139). Hence, strain 139 may be proposed as the best candidate for use as a starter in MLF.

*O. oeni* strains have a compact genome of 1.8 Mb and several metabolic pathways related to growth in enological environments, including MLF and aroma production (Mills et al., 2005; Makarova et al., 2006; Makarova and Koonin, 2007). Furthermore, its compact genome most likely reflects a high level of organization and simplicity (Jara and Romero, 2015; Sternes and Borneman, 2016). This genomic organization may be the basis for its adaptation to the wine environment (Zé-Zé et al., 1998, 2000; Mills et al., 2005). Interestingly, the analyses of these genomes using ANIb, DDH and OrthoANI revealed 139 consistent differences from the autochthonous strains when the distance between genomes was calculated by aligning the whole sequences. According to Thompson et al. (2013), these isolates and reference strains (VP41 and PSU-1) shared more than 95% ANIb and hence could be considered the same species. A similar observation including more than 30 *O. oeni* genomes was also reported by Campbell-Sills et al. (2015).

A report by Borneman et al. (2010) compared the genomes of three *O. oeni* strains (PSU-1, BAA1163, and AWRIB429). These strains shared conserved genes corresponding to 52% of the observed ORFs. These authors claimed that this conserved region could be considered the core genome. A similar result was reported by Campbell-Sills et al. (2015) and Sternes and Borneman (2016), but these studies included more CDSs due to differences in the orthologous calculation. Borneman et al. (2010) posited that unique ORFs associated with bacteriophage-derived sequences or glycosyl hydrolases might be key from a winemaking perspective, because these ORFs might contribute to aromatic compound formation through the cleavage of the sugar moiety from the non-volatile (and therefore aroma-less) glycosidic precursors present in grape juice. These analyses suggest that genomic variation may be the key to ascertaining the phenotypic differences between *O. oeni* strains. In this context, our data showed that the Chilean strain contained 21–28 unique genes per strain related to metabolism and transport, some of which possibly explained some of the technological properties of the bacteria. Similarly, Campbell-Sills et al. (2015) reported that *O. oeni* isolates from Champagne showed 27 unique genes that might be related to technical properties. Taken together, the technical properties, unique characteristics and capacity for local adaptation of some LAB could provide the basis for obtaining suitable strains to serve as inocula in future products and contribute to the typicity of Chilean wines.

Several enzymes from *O. oeni* may contribute to the aromatic profile of the wine during MLF, including β-glucosidase, citrate lyase, esterases, proteases, and peptidases (Mtshali et al., 2010; Michlmayr et al., 2012; Cappello et al., 2017). The activity of some of these enzymes may be modulated by enological parameters, such as the pH, temperature, ethanol, or glucose and fructose concentrations (Grimaldi et al., 2005). Furthermore, Olguín et al. (2011) demonstrated that the expression of the β-glucosidase gene (*bgl*) in *O. oeni* might be induced by a moderate ethanol concentration.

Citrate metabolism is involved in the production of compounds, such as diacetyl, acetoin, butanediol and acetate, which are important for the wine aroma (Olguín et al., 2009). Diacetyl is the most important aroma compound during MLF (Cappello et al., 2017). Additionally, some genes involved in citrate metabolism have been shown to provide metabolic traits to different strains (Olguín et al., 2009). The inter-strain comparison of the transcriptional levels of genes involved in citrate metabolism (*ack*A and *als*D) revealed that the strains had different metabolic features.

Esters are a key group of volatile compounds that can contribute to the wine aromatic profile (Swiegers et al., 2005). These compounds depend on the activity of esterases (Cappello et al., 2017). Sumby et al. (2013) showed that two purified *O. oeni* esterases (EstA2 and EstB28) had two activities (synthesis and hydrolysis) that suggested the contribution of *O. oeni* to the wine aroma profile.

The contribution of specific *O. oeni* strain to the organoleptic properties of wine may affect flavor formation depending on the wine parameters (Cappello et al., 2017). The selection of bacterial strains for MLF should consider the potential to improve the wine typicity. In this context, Chilean isolates showed different contents of genes encoding enzymes contributing to the aromatic profile; among them, strain 139 presented a higher number of glucosidases and promising enological properties and thus might be proposed as the best candidate for use as a starter in MLF.

To the best of our knowledge, this study is the first report to focus on both the genetic and technological characterization of *O. oeni* strains in Chile. This study reported that genetic (MLST) and genomic tools (ANI) might reveal the differences between commercial and autochthonous *O. oeni* strains. Similarly, autochthonous *O. oeni* strains showed some advantages in terms of technological properties. Thus, future studies should focus on determining the potential relationships between the phylogenetic and phenotypic characteristics of *O. oeni* strains; these results could help identify the effect of environmental conditions on the genetic content and evolution of the species. Furthermore, these analyses may provide useful information for the selection of strains with better industrial performances.

## Author contributions

JR and CJ: designed of the work; CI: data collection, data analysis, and interpretation; MR: Genetic and genomic analyses; CJ: drafting the article; JR: critical revision and edition of the article; CJ, CI, JR: Final approval of the version to be published.

### Conflict of interest statement

The authors declare that the research was conducted in the absence of any commercial or financial relationships that could be construed as a potential conflict of interest.
