# Supplementary material for: Oenococcus oeni in Chilean Red Wines: Technological and Genomic Characterization
Source: Front Microbiol. 2018 Feb 14;9:90. doi: 10.3389/fmicb.2018.00090 (PMC5817079; doi:10.3389/fmicb.2018.00090)

Supplementary Material

Technological properties of autochthonous *O. oeni* isolates compared to the commercial strain (VP41).

The ability of the isolates to degrade two concentrations of malic acid (mg/L);
(a) 1,5 mg/L malic acid concentration
(b) 4 mg/L malic acid concentration.

(a)

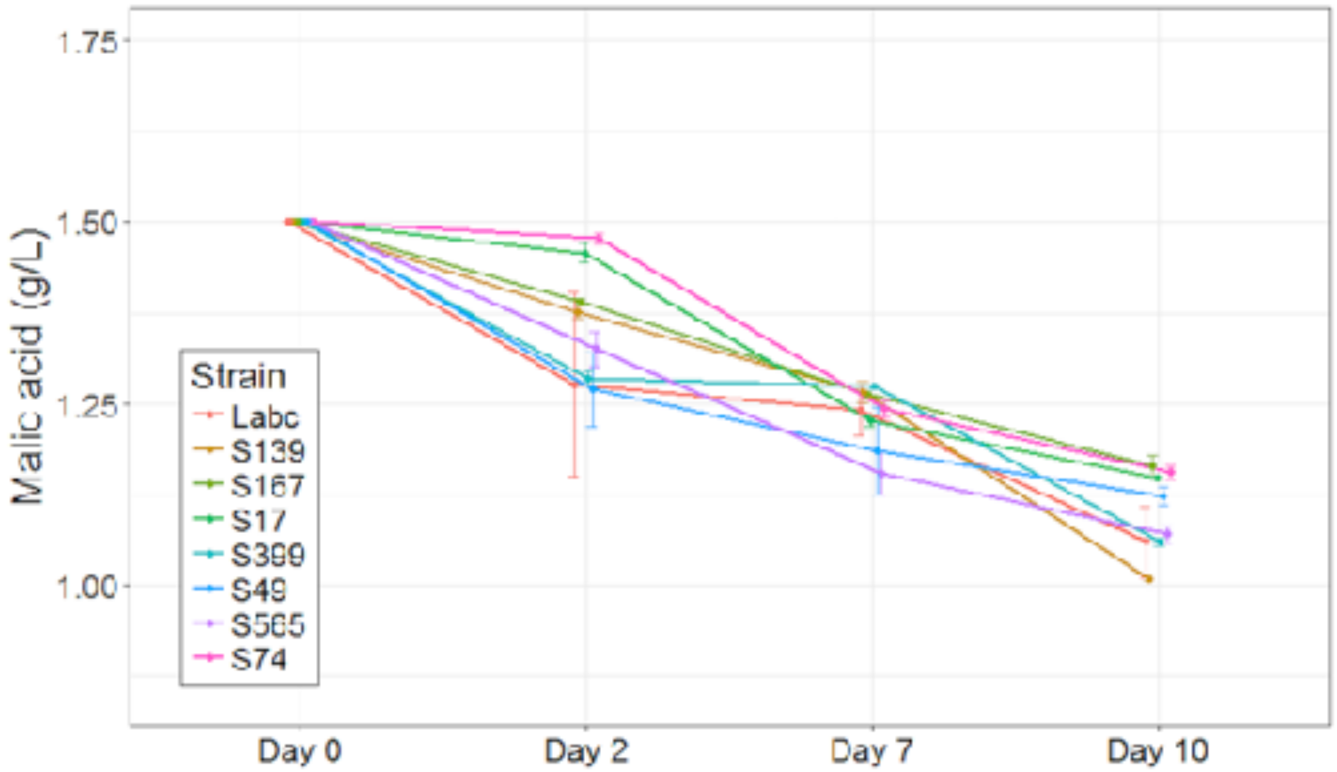

(b)

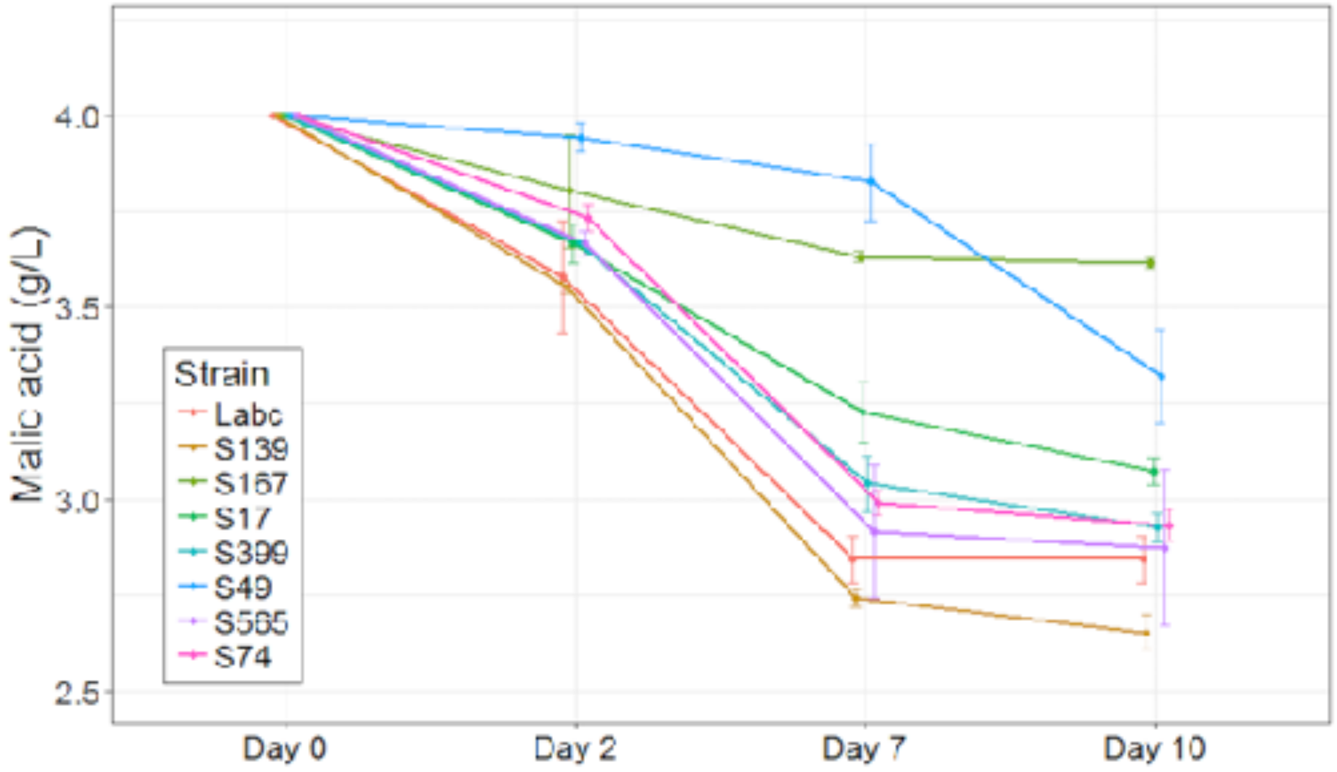

The growth of *O. oeni* isolates in two ethanol concentrations (scale log_{10});
(c) 12% v/v ethanol
(d) 15% v/v ethanol

(c)

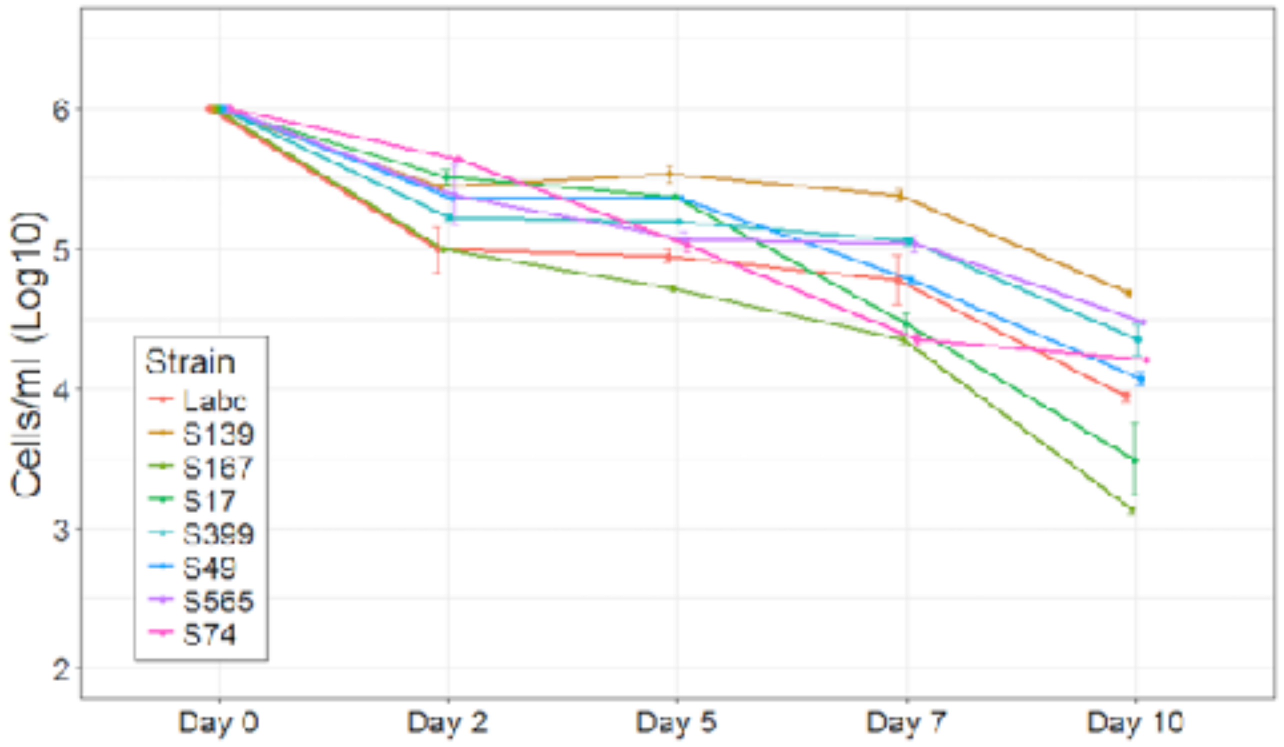

(d)

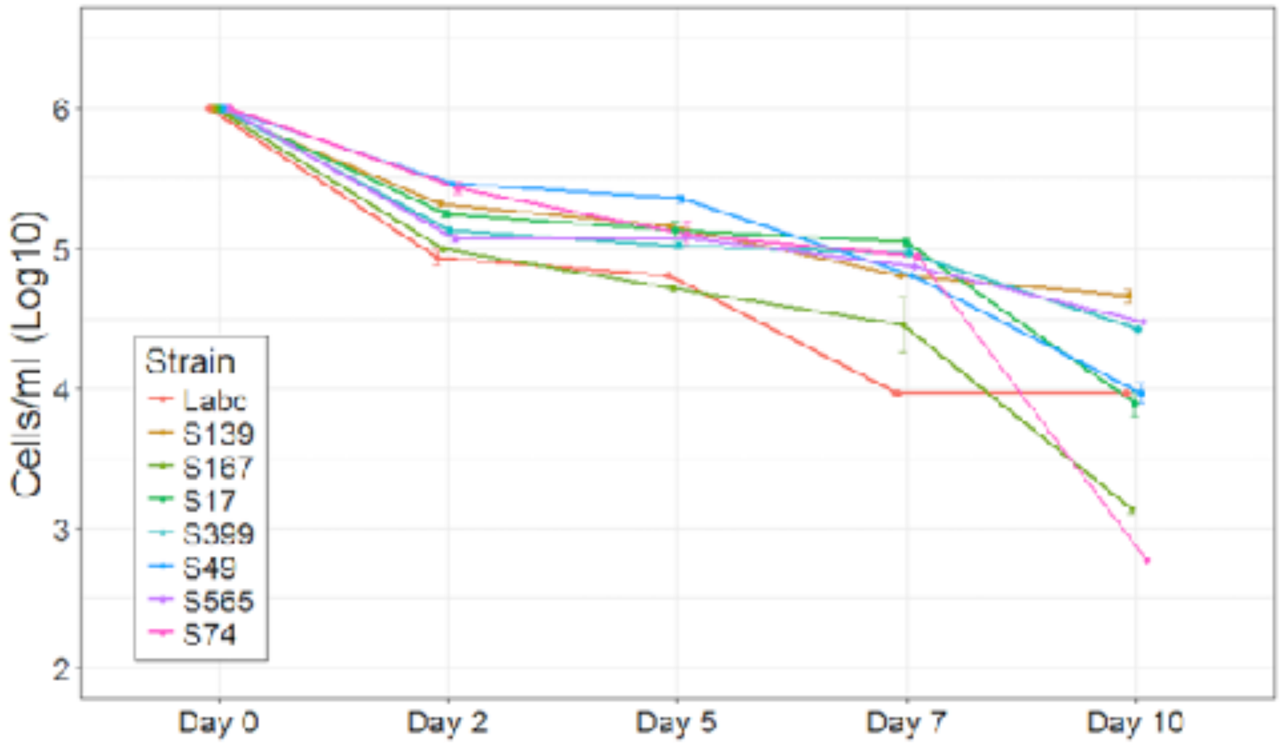

The growth and tolerance of *O. oeni* isolates at two pH values (scale $\log_{10}$);
(e) pH 3.1
(f) pH 3.6.

(e)

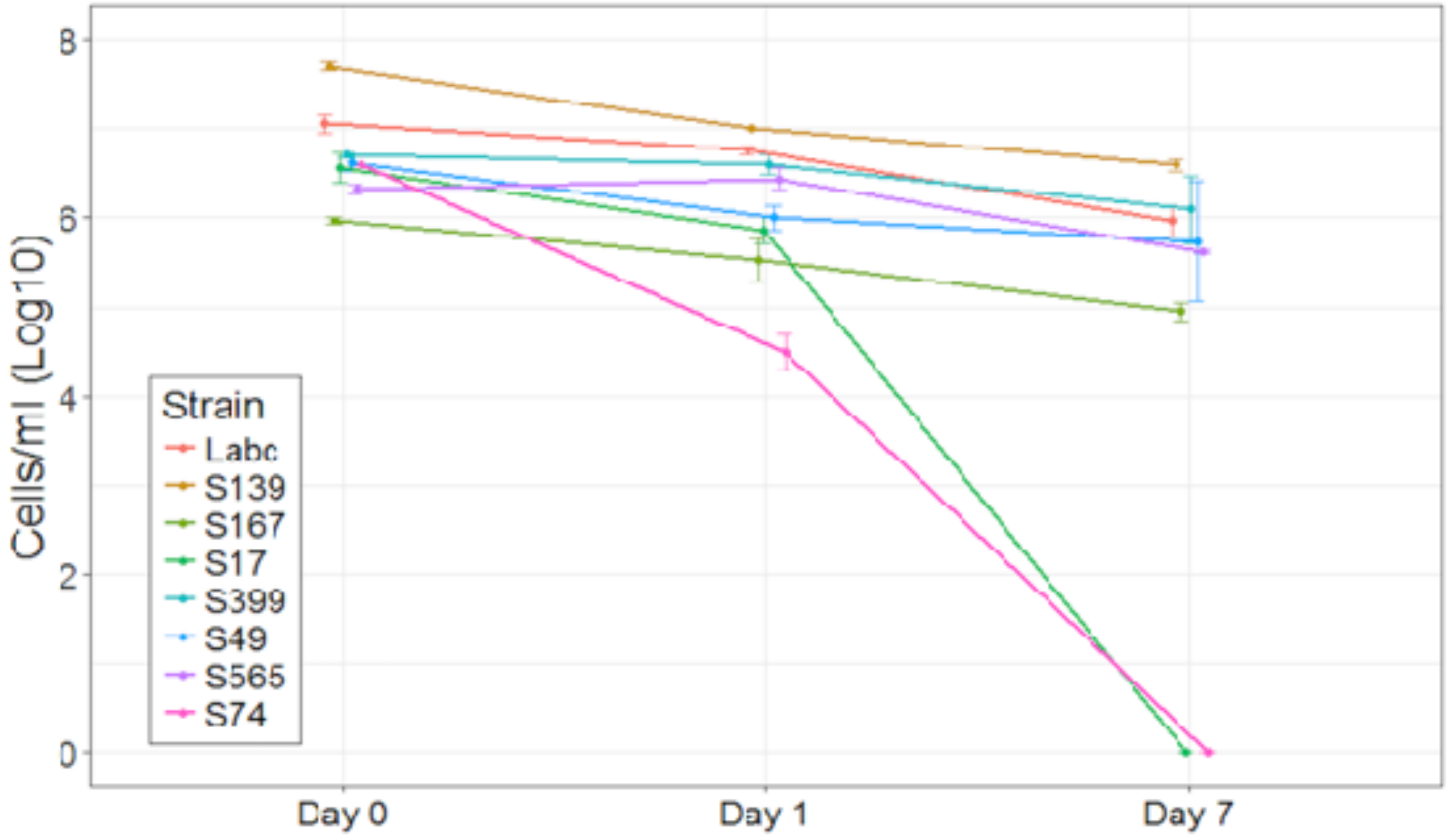

(f)

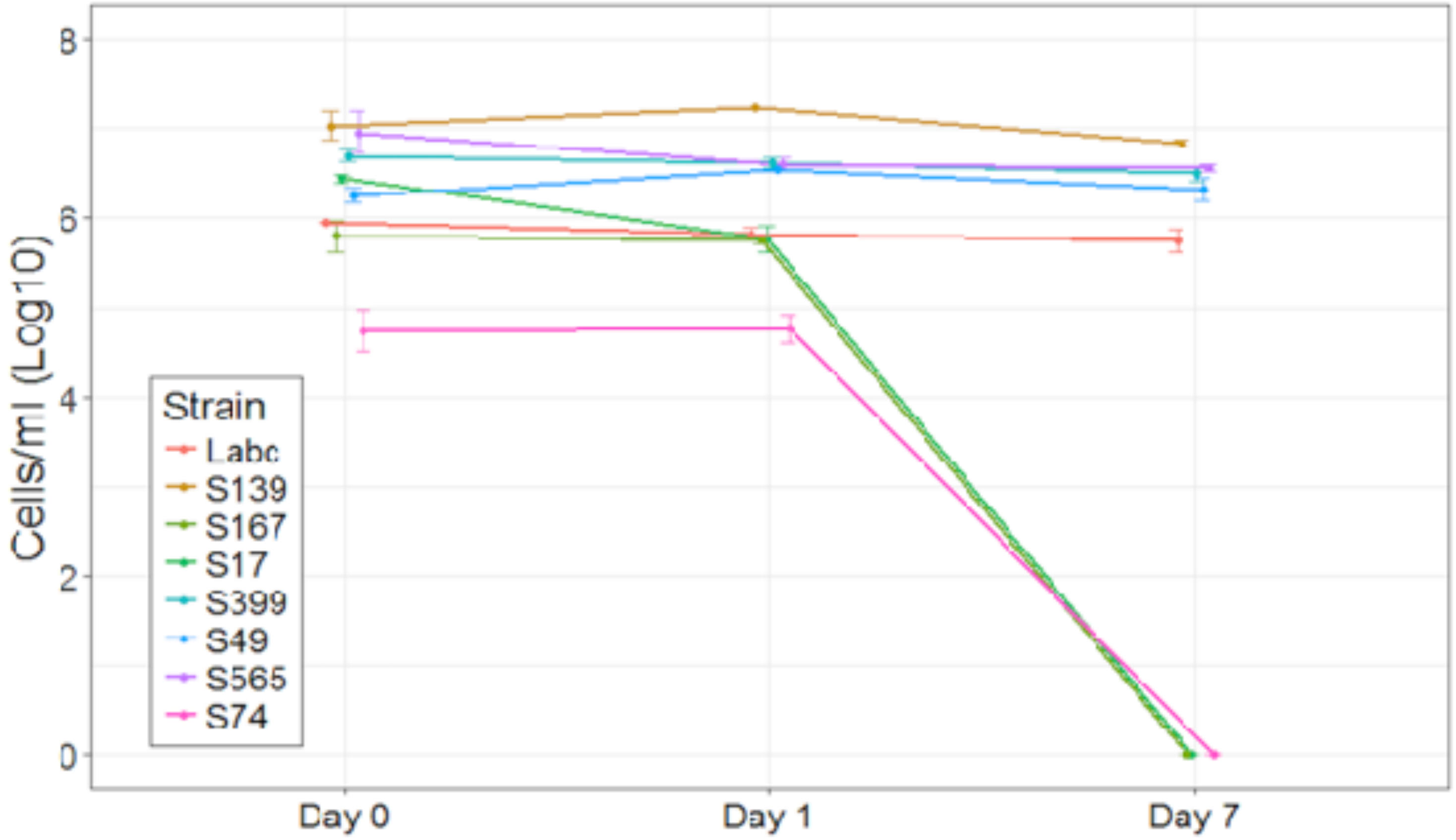

The growth and tolerance of *O. oeni* isolates to two SO_2 concentrations (scale $\log_{10}$);
(g) 0 ppm free SO_2.
(h) 80 ppm free SO_2.

(g)

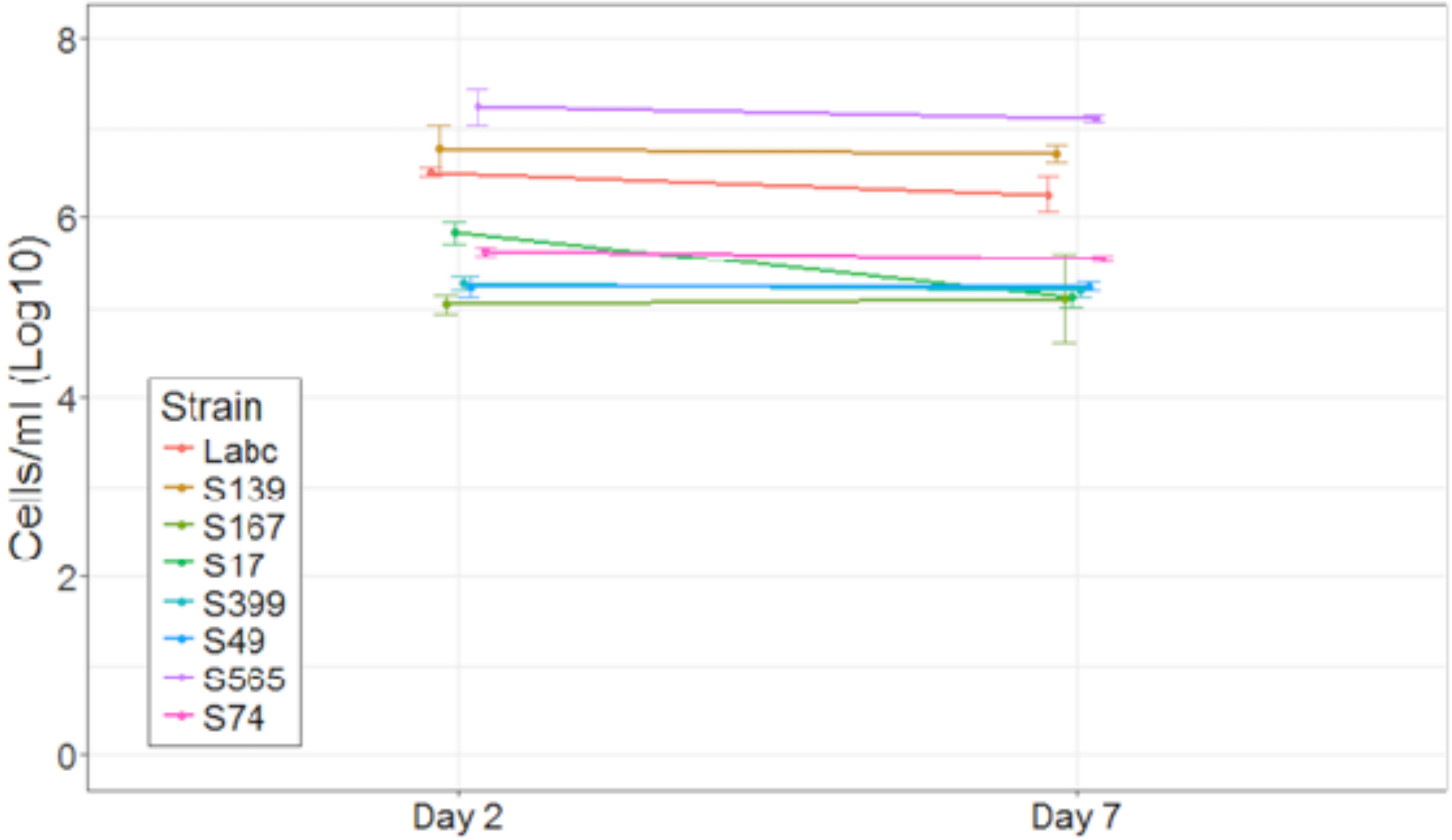

(h)

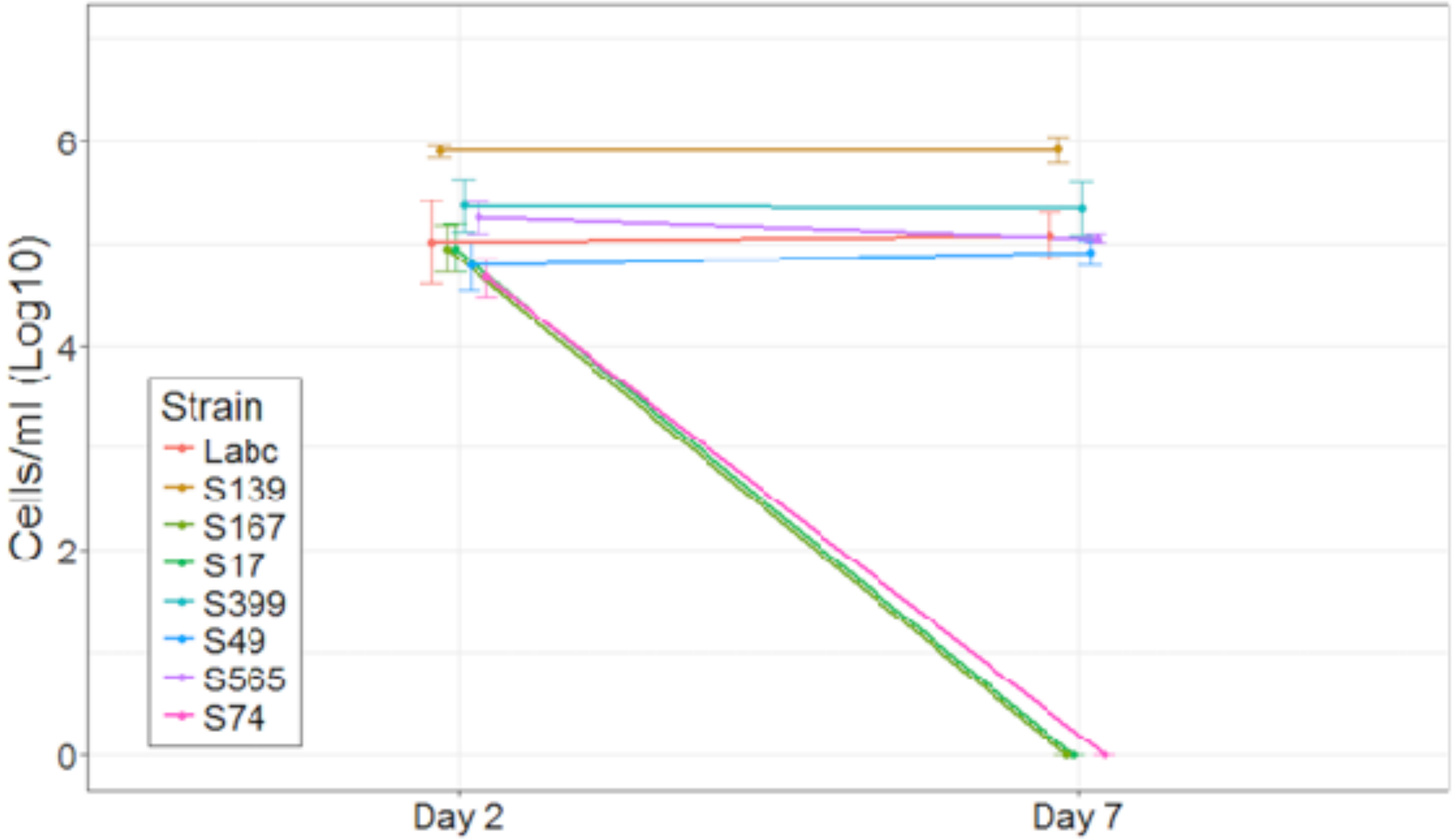

Supplement: Supplementary file 1 [file Presentation1.PDF]
