# Supplementary material for: Oenococcus oeni in Chilean Red Wines: Technological and Genomic Characterization
Source: Front Microbiol. 2018 Feb 14;9:90. doi: 10.3389/fmicb.2018.00090 (PMC5817079; doi:10.3389/fmicb.2018.00090)
Supplement: Supplementary file 2 [file Presentation2.PDF]

Supplementary table S1. Presence or absence of *rec*P gene in *Oenococus oeni* genomes different origins.

Origin	Genomes reported	Genomes analyzed	Name	Presence of *recP* gene	Absence of *recP* gene	Refernce/ BioProject
France (Bordeaux)	1	1	ATCC BAA-1163	-	1	PRJNA16668
Argentine	1	1	X2L	-	1	PRJNA203385
Italy	4	4	OT 3	1	-	PRJNA253367
			OT 4	1	-	PRJNA253853
			OT 5	1	-	PRJNA253780
			OT 25	1	-	PRJNA253854
Chile	4	4	49; 139; 399; 565	-	4	PRJNA282732
Australia	1	1	DMS 20252	1	-	PRJNA133375
USA	1	1	DMS 20252	1	-	PRJNA165413
Italy	2	2	OM 22	1	-	PRJNA253810
			OM 27	1	-	PRJNA245799
France	11	11	S 11	1	-	PRJNA229592
			S 12	-	1	PRJNA229617
			S 13	1	-	PRJNA229642
			S 14	1	-	PRJNA229684
			S 15	1	-	PRJNA229729
			S 19	-	1	PRJNA229731
			S 22	-	1	PRJNA229732
			S 23	1	-	PRJNA229733
			S 25	1	-	PRJNA229735
			S 28	1	-	PRJNA229736
			S 161	-	1	PRJNA229730
France	14	14	CRBO: 14211; 14205	2	-	PRJNA297331
			CRBO: 11105; 14194; 14195; 14196; 14198; 14200; 14203; 14206; 14207; 14210; 14213; 14214	-	12	
France	25	25	IOCB 0205	1	-	PRJNA229526
			IOCB 0501	1	-	PRJNA229527
			IOCB 0502	-	1	PRJNA229528
			IOCB 0607	-	1	PRJNA229529
			IOCB 8417	-	1	PRJNA229533
			IOCB 0608	-	1	PRJNA229531
			IOCB 9517	-	1	PRJNA229543
			IOCB 9803	1	-	PRJNA229544
			IOCB 9805	-	1	PRJNA229545
			IOCB S277	1	-	PRJNA229565
			IOCB B16	1	-	PRJNA229547
			IOCB B10	1	-	PRJNA229546
			IOCB CiNe	-	1	PRJNA229552
			IOCB C23	-	1	PRJNA229549
			IOCB L65 2	1	-	PRJNA229557
			IOCB C28	-	1	PRJNA229550
			IOCB L40 4	-	1	PRJNA229556
			IOCB S436a	1	-	PRJNA229566
			IOCB C52	1	-	PRJNA229551
			IOCB 1491	-	1	PRJNA229532
			IOCB VF	1	-	PRJNA229569
			IOCB L18 3	1	-	PRJNA229553
			IOCB L26 1	1	-	PRJNA229554
			IOCB S450	1	-	PRJNA229567
			IOCB 9304	-	1	PRJNA229534
Australia	149	50	AWRIB 304	1	-	PRJNA89139
			AWRIB 318	1	-	PRJNA133377
			AWRIB 419	1	-	PRJNA169939
			AWRIB 418	1	-	PRJNA169938
			AWRIB 422	1	-	PRJNA169940
			AWRIB 548	1	-	PRJNA169941
			AWRIB 553	1	-	PRJNA169942
			AWRIB 576	1	-	PRJNA169944
			AWRIB 568	1	-	PRJNA169943
			AWRIB 202	1	-	PRJNA89141
			AWRIB 429	-	1	PRJNA38663
			AWRIB: 1059; 899; 714; 435; 1116; 130; 136; 216; 330; 342; 346; 151; 335; 338; 424; 430; 441; 446; 462; 438; 447; 490; 583; 503; 634; 661; 712; 713; 819; 853; 863; 888; 121; 324; 867; 900; 950; 710; 873	-	39	PRJNA304199
Total	213	114		40	74	
